# Hilbert-Huang Transform Embedded Self-Attention Neural Network for EEG-based major depressive disorder vs. healthy controls classification

**DOI:** 10.3389/fpsyt.2025.1658918

**Published:** 2025-11-06

**Authors:** Junxian Chen, Kaikun Tian, Yu Ye, Jiaming Liu

**Affiliations:** 1 Suzhou Industrial Park Institute of Services Outsourcing, School of Information Engineering, Suzhou, Jiangsu, China; 2 School of Physics and Electronic Science, Hubei Normal University, Huangshi, China; 3 Department of Radiology, The Central Hospital of Huangshi City, Huangshi, China

**Keywords:** classification, depression, EEG, Hilbert-Huang Transform, Self-Attention Neural Network

## Abstract

This paper proposes a novel approach for distinguishing Major Depressive Disorder (MDD) patients from healthy controls (HC), namely depression screening, using EEG signals, where the Hilbert-Huang Transform (HHT) is integrated into a Self-Attention neural network (HHT-SANN). The incorporation of the HHT enhances the model’s time-frequency analysis capabilities and allows for more effective nonlinear processing of the EEG data. By embedding the HHT within the self-attention module, the model captures intricate temporal and spectral patterns that are critical for accurate depression classification. We evaluated our method on a clinical EEG dataset comprising 34 MDD patients and 30 healthy controls from the Hospital of Universiti Sains Malaysia. Experimental results indicate that the proposed method achieves an accuracy of 98.78%, sensitivity of 99.23%, and specificity of 98.27%, outperforming traditional models and offering a more robust solution for depression detection. This work contributes to advancing the field of neuroinformatics by providing a more interpretable and effective model for mental health diagnostics based on EEG data.

## 1 Introduction

Depression is a pervasive and debilitating psychiatric disorder affecting hundreds of millions worldwide, with those experiencing severe major depressive disorder (MDD) at particularly high risk of suicidal ideation. Objective and reliable screening methods are therefore critical for early intervention and improved outcomes (1). However, traditional diagnostics—relying on clinician assessments and self-report questionnaires—are vulnerable to bias and inconsistency, fueling the search for quantitative biomarkers. In this context, compared with fMRI (2, 3) and structural MRI (4), electroencephalography (EEG) has emerged as a noninvasive, cost-effective modality with exceptional temporal resolution, adept at capturing the rapid neural dynamics underlying depressive pathology.

The efficacy of EEG-based diagnosis, however, hinges critically on two interdependent pillars: the capability of the classifier and, just as importantly, the appropriateness of the feature extraction methodology for uncovering the neural mechanisms specific to the disorder. EEG signals are inherently non-linear and non-stationary, reflecting the complex, dynamic nature of brain activity in MDD. Consequently, the choice of feature extraction is paramount. A diverse array of approaches has been explored in the literature to capture these aberrant neural patterns. For instance, studies have investigated functional connectivity and network properties, such as spectral coherence (5), domain-specific connectivity (6), and global EEG connectivity (7), which aim to reveal disorganization in large-scale brain networks associated with MDD. Others have focused on complexity measures and nonlinear dynamics to quantify the irregularity of neural signals (8), or have utilized connectivity features derived from EEG to predict treatment outcomes (9, 10).

While these methods provide valuable insights, many conventional feature extraction techniques, including those based on predefined linear transforms or hand-crafted metrics, may not fully adapt to the non-stationary and nonlinear characteristics of EEG in depression. This underscores a pressing need for methods that can effectively and adaptively model the time-varying, nonlinear dynamics of brain activity in depressive individuals. For example, Mumtaz et al. (11) proposed a machine learning method using pre-treatment EEG to predict SSRI antidepressant outcomes in MDD. Subsequently, Ke et al. developed an AutoML-based dual-CNN model for real-time EEG classification in brain e-health, addressing static models and computational complexity with autonomously optimized hyperparameters, achieving high accuracy for MDD and significantly outperforming CapsuleNet and ResNet-16 (12). More recently, a novel TanhReLU-based CNN was proposed to address MDD diagnosis challenges using EEG data, with the hybrid activation function combining Tanh and ReLU properties to mitigate gradient vanishing and overfitting in EEG pattern recognition (13). Wang et al. (14) enhanced MDD/BD classification by integrating clinical and EEG data from 400 patients, using feature engineering and ML models to improve accuracy, address overfitting, and highlight EEG’s diagnostic value for precision psychiatry.

The primary motivation for integrating HHT into a deep learning framework stems from its inherent suitability for analyzing the non-linear, non-stationary brain dynamics characteristic of MDD. Unlike methods relying on predefined basis functions, HHT’s fully adaptive decomposition into Intrinsic Mode Functions (IMFs) offers a physiologically plausible representation of EEG signals, enabling direct extraction of instantaneous frequency and amplitude components beyond linear assumptions. Our core theoretical contribution lies in conceptualizing HHT not as a fixed pre-processor, but as an integrated layer within an end-to-end network. This allows the model to learn task-specific time-frequency representations directly from raw data, optimizing feature extraction for MDD identification. Thus, this work bridges theoretically-grounded signal processing for non-stationary data and deep learning’s pattern recognition capabilities.

While HHT provides meaningful time-frequency representations, it remains under-explored as an integrated component in deep learning frameworks (15). Prior implementations often treated HHT as a static preprocessing step, limiting end-to-end optimization. To address this gap and better link neural mechanism-informed feature extraction with classification power, we propose the novel Hilbert-Huang Transform Embedded Self-Attention Neural Network (HHT-SANN).

In HHT-SANN, the HHT is embedded as a network layer to learn intrinsic time-frequency components dynamically. It is complemented by a Squeeze-and-Excitation (SE) module that adaptively recalibrates feature channels, and a self-attention mechanism that captures global dependencies in time-frequency representations. These components collectively form an end-to-end system that unifies advanced feature extraction rooted in MDD’s neural substrate with high-performance classification for EEG-based screening.

To summarize, the main contributions of this study are as follows:

1. We propose the first deep learning model that integrates the Hilbert-Huang Transform as a layer for EEG-based depression classification.
2. We introduce a novel combination of HHT, self-attention, and channel-wise excitation, enabling the model to learn adaptive and interpretable time-frequency representations.
3. We validate our method on real-world EEG datasets and demonstrate its superiority over conventional and state-of-the-art approaches in terms of classification accuracy and robustness.

## 2 Methodology

### 2.1 Dataset

The EEG dataset used in this study was acquired at the Hospital of Universiti Sains Malaysia and comprises recordings from 34 patients with Major Depressive Disorder (MDD: 17 males, mean age 40.3 ± 12.9 years) and 30 healthy controls (21 males, mean age 38.2 ± 15.6 years) (11). All participants underwent 5 min of eyes-closed and 5 min of eyes-open resting-state EEG, recorded via 20 scalp electrodes (Fp1, Fp2, F3, F4, F7, T3, T5, C3, C4, Fz, Cz, Pz, F8, T4, T6, P3, P4, O1, O2, ECG) placed according to the international 10–20 system at 256 Hz. Exclusion criteria included psychotic symptoms, pregnancy, alcohol/substance use, smoking, or epilepsy; controls were screened to confirm the absence of neurological or psychiatric conditions. EEG data were preprocessed in BESA to remove artifacts, and two-minute artifact-free segments from each resting condition were extracted using a 1,024-sample (4 s) sliding window, yielding 18,442 total epochs (9,789 MDD, 8,653 HC).

### 2.2 Hilbert Huang Transform Layer

**Figure 1:**
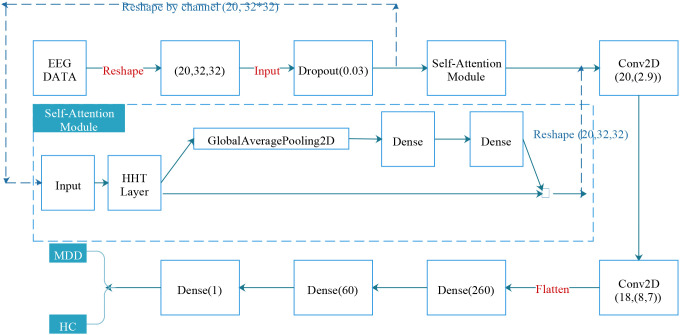
Model architecture.

This section introduces our proposed model architecture, which integrates the Hilbert-Huang Transform (HHT) into a deep neural network for EEG-based depression screening. Our architecture consists of three main components: a Hilbert Transform Layer, and a Squeeze-and-Excitation (SE) block, which forms the Self-Attention module. The overall architecture illustrated in **Figure 1** is designed to learn discriminative, non-linear time-frequency features from raw EEG inputs in an end-to-end fashion.

In this approach, the HHT layer is implemented as a neural network layer that applies Empirical Mode Decomposition (EMD) followed by the Hilbert Transform to the input EEG signal. Each EEG signal *x*(*t*) for each channel (20, 32*32) is decomposed into a set of Intrinsic Mode Functions (IMFs) (Equation 1):

(1) $$x(t)=\sum_{i=1}^{N}{\text{IMF}}_{i}(t)+r(t)$$

where *N* is the number of IMFs and *r*(*t*) is the residual signal.

The Hilbert Transform is then applied to each IMF to obtain its instantaneous amplitude and frequency. The analytic signal *h*(IMF*_i_*(*t*)) is defined as Equation 2:

(2) $$h({\text{IMF}}_{i}(t))={\text{IMF}}_{i}(t)\cdot \text{exp }(j\cdot \theta_{i}(t))$$

The instantaneous frequency $f_{i}(t)$ is computed as Equation 3:

(3) $$f_{i}(t)=\frac{1}{2\pi }\frac{d\theta_{i}(t)}{dt}$$

The output of the HHT layer is a time-frequency feature matrix $X_{\text{HHT}}$ that incorporates both channel and temporal information from the EEG signal.

### 2.3 Squeeze-and-Excitation block with HHT

To enhance channel-wise representations, a Squeeze-and-Excitation (SE) block is incorporated after the HHT layer. Let $F_{\text{HHT}}\in {\mathbb{R}}^{T\times C^{\prime }}$ denote the output from the HHT layer, where $C^{\prime }$ represents the expanded feature dimension after IMF concatenation, and T is the number of time points.

**Squeeze step:** Global average pooling is applied across the temporal dimension to generate a channel descriptor (Equation 4):

(4) $$z_{c}=\frac{1}{T}\sum_{t=1}^{T}F_{\text{HHT}}(t,c)$$

**Excitation step:** The descriptor $z\in {\mathbb{R}}^{C^{\prime }}$ is passed through two fully connected layers with a ReLU activation followed by a sigmoid function (Equation 5):

(5) $$s=\sigma (W_{2}\cdot \text{ReLU}(W_{1}\cdot z))$$

where $W_{1}\in {\mathbb{R}}^{\frac{C^{\prime }}{r}\times C^{\prime }}$, $W_{2}\in {\mathbb{R}}^{C^{\prime }\times \frac{C^{\prime }}{r}}$, and *r* is the reduction ratio controlling the bottleneck compression. The sigmoid function σ(·) ensures the output values lie in the range (0, 1).

**Scale:** The excitation vector s is broadcast and multiplied element-wise with the original feature map $F_{\text{HHT}}$ to generate the recalibrated feature map ${\tilde{F}}_{\text{SE}}$ in Equation 6:

(6) $${\tilde{F}}_{\text{SE}}(t,c)=s_{c}\cdot F_{\text{HHT}}(t,c)$$

This operation allows the network to emphasize informative features and suppress less relevant ones by adaptively adjusting the importance of each channel.

## 3 Results

The experiments conducted in this section serve as a validation and assessment of the classification performance of the proposed model. Initially, we describe the experimental platform utilized for these assessments. Finally, the classification effectiveness of the TanhReLU-based Convolutional Neural Network (CNN) is evaluated using metrics such as accuracy, sensitivity, and specificity (see Section 3.2). The experiments were executed on a desktop equipped with an Intel i7 CPU operating at 3.33GHz, an Nvidia RTX 1080Ti GPU, 32GB RAM, and running Windows 10. This system configuration ensured consistent testing conditions throughout the experiments.

### 3.1 Feature importance analysis

**Figure 2:**
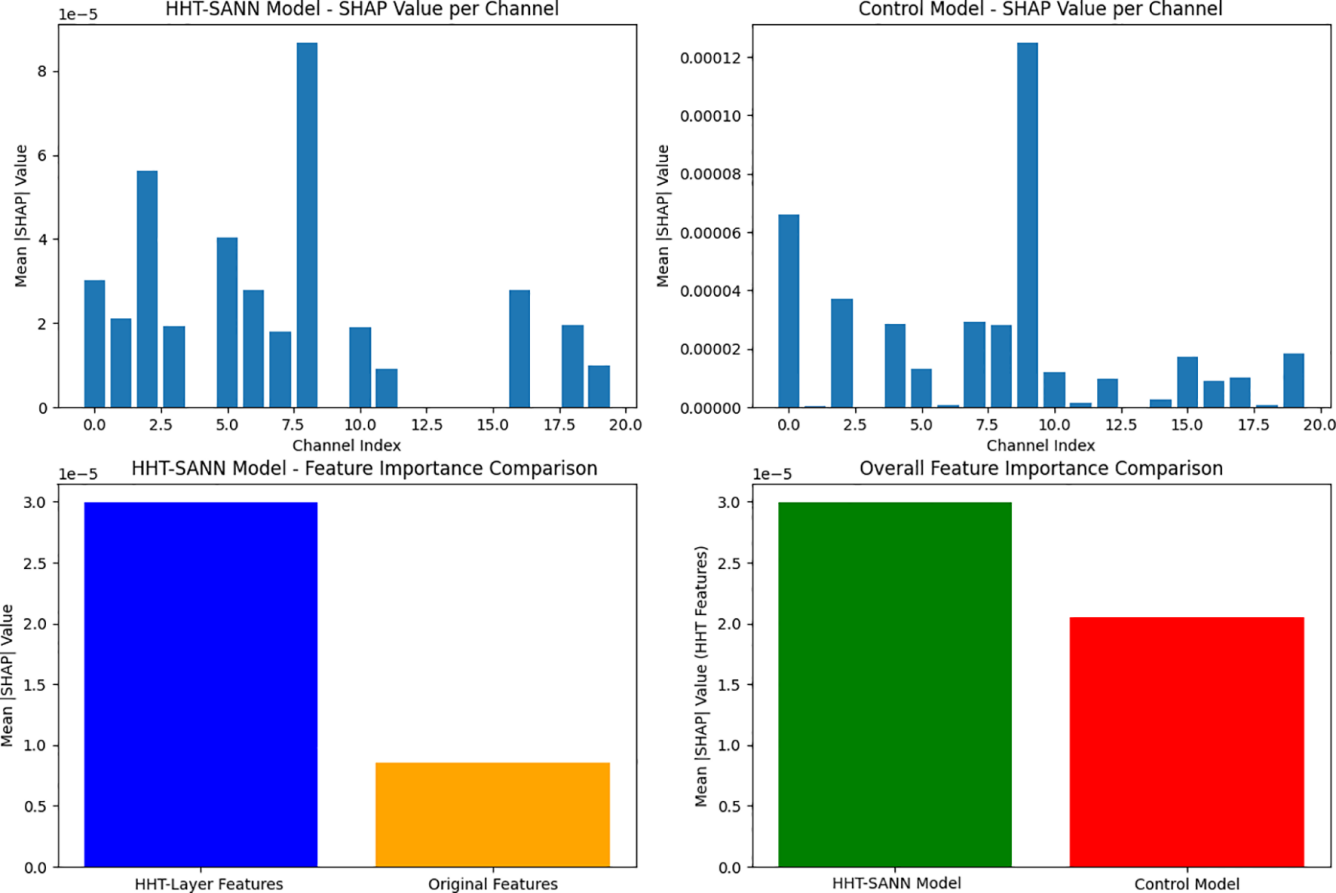
Feature importance comparison.

To quantify the contribution of Hilbert-Huang Transform (HHT) features compared to original signal features to model decision-making, this study employed the SHAP (SHapley Additive exPlanations) method (16, 17) for feature importance analysis, which illustrated in **Figure 2**. The specific procedure is as follows: using the KernelSHAP explainer (applicable to any model), one training sample was randomly selected as the background, and calculations were performed on 10 independent test samples. Feature importance was quantified by calculating the mean absolute SHAP value (|SHAP|) for each channel, with specific separation of HHT feature channels (containing amplitude and frequency information) and original signal channels. Finally, the contributions were compared by calculating the importance ratio R (R = Mean HHT Feature Importance/Mean Original Feature Importance). The experimental results show that HHT features exhibit significantly higher importance than original features, with the importance ratio R reaching as high as 3.52 2. This fully demonstrates that HHT features play a dominant role in model decision-making.

### 3.2 Performance on identifying MDD

**Figure 3:**
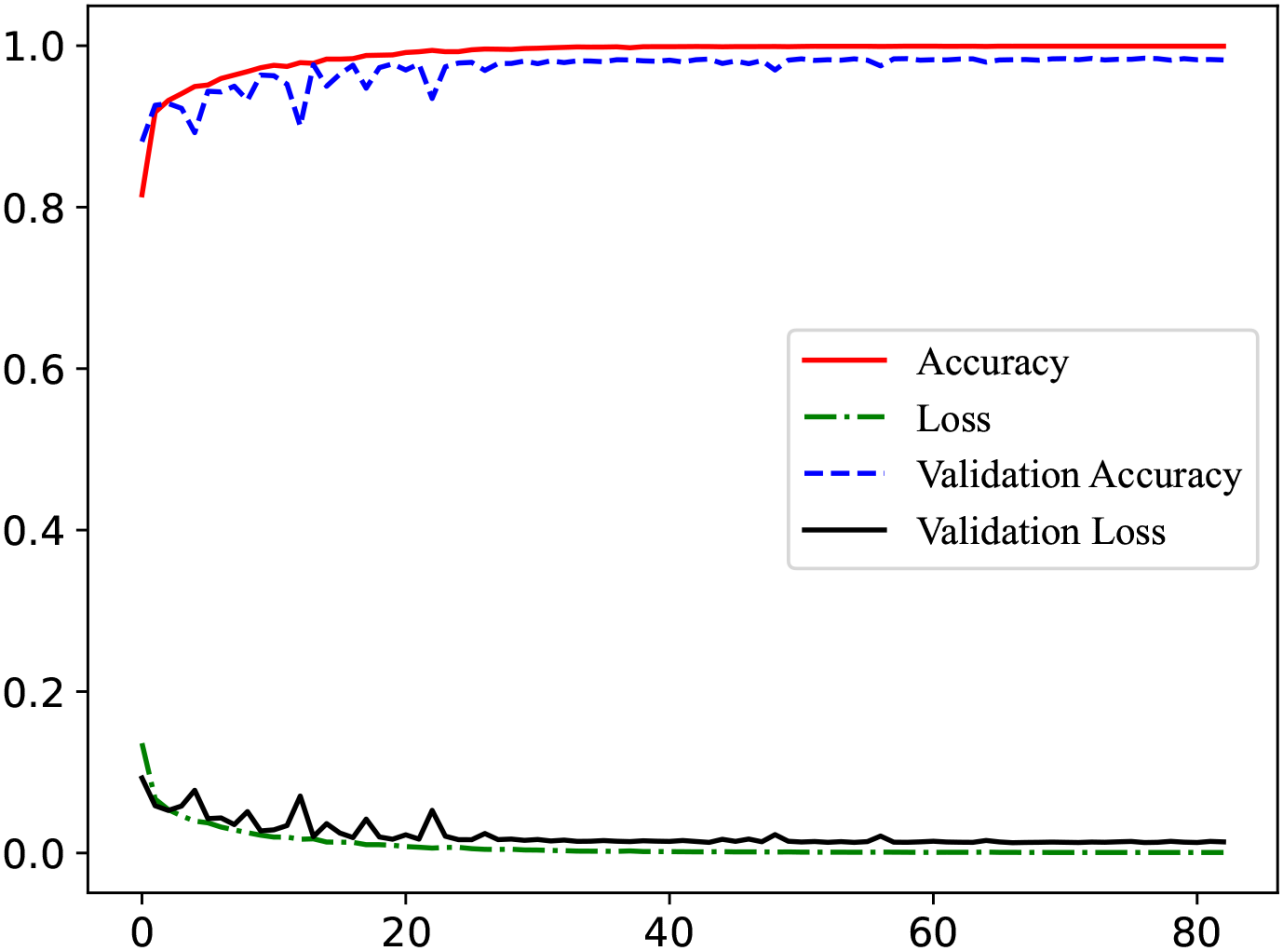
Learning curve for classifying MDD.

**Figure 3** presents the model’s learning curves. As shown, the training accuracy (red line) rises rapidly within the first few epochs and approaches 1 (nearly 100%), indicating that the model has sufficient capacity to fit the training data well. Meanwhile, the validation accuracy (blue line) also increases quickly at an early stage and eventually becomes close to the training accuracy, demonstrating good generalization to unseen data. Both the training loss and validation loss (green and black dashed lines, respectively) decrease steadily and converge toward zero, with the training and validation accuracies remaining highly consistent. Therefore, the model does not exhibit any obvious signs of underfitting or overfitting.

Finally, the classification performance of the proposed model was assessed on the designated test set using a Leave-One-Subject-Out cross-validation protocol. It achieved an impressive accuracy of 98.78%, along with a sensitivity of 99.23% and a specificity of 98.27%, as summarized in **Table 1**. Among the baseline approaches, CapsuleNet demonstrates strong specificity (99.23%) but comparatively low sensitivity (89.01%), leading to a moderate overall accuracy of 94.42%. ResNet-16 yields the lowest performance across all metrics, particularly in specificity (74.79%), which significantly reduces its classification reliability (82.26% accuracy). The MLRW method shows balanced but suboptimal results with 95% sensitivity and 80% specificity.

**Table 1:** Comparative analysis of the performance between the proposed methodology and existing state-of-the-art approaches.

Approach	Sensitivity (%)	Specificity (%)	Accuracy (%)
CapsuleNet (13)	89.01	99.23	94.42
Resnet-16 (13)	88.9	74.79	82.26
MLRW	95	80	87.5
Our(without HHT)	98.05	**99.25**	98.61
Our	**99.23**	98.27	**98.78**

Bold indicates the optimal value for each performance index.

Our proposed model, both with and without the Hilbert-Huang Transform (HHT), outperforms all baselines. The ablated version (without HHT) achieves a notable accuracy of 98.61% and the highest specificity (99.25%), indicating a strong ability to correctly identify negative cases. However, the full version of our model (with HHT) achieves the highest sensitivity (99.23%) and the best overall accuracy (98.78%), demonstrating improved detection of positive cases and overall classification robustness.

This comparison highlights the benefit of incorporating the HHT module, which effectively enhances the model’s sensitivity while maintaining high specificity. The trade-off between the two versions of our model reflects a subtle balance between reducing false negatives and false positives, which is crucial in clinical applications where missed detections can have serious consequences.

## 4 Discussions and conclusions

Importance of HHT: Quantitatively, our model achieved 98.78% test accuracy (sensitivity 99.23%, specificity 98.27%), outperforming conventional baselines (see **Table 1**). In particular, incorporating the HHT layer boosted sensitivity from 98.05% to 99.23%, at a minimal cost to specificity. The SHAP feature-importance analysis further confirms the impact of HHT-based features: the mean absolute SHAP value for HHT-derived channels was 3.52 times that of the original signal channels. In other words, HHT-driven time-frequency components dominate the model’s decision-making, highlighting that the adaptive nonlinear features extracted by HHT are far more informative than raw time-domain EEG samples. This dominance of nonlinear features is consistent with prior studies showing that nonlinear EEG characteristics (e.g. asymmetry, entropy measures) are powerful biomarkers of depression. By embedding HHT as a network layer, our network learns task-specific intrinsic mode functions (IMFs) directly in the time domain, which aligns with the known strength of HHT: it is explicitly designed for nonstationary, nonlinear data and preserves the instantaneous frequency content of the signal.

Limitations and future works: Nevertheless, there are limitations. The high training accuracy warrants caution about overfitting to the specific dataset, even though validation results were strong. Like most EEG-depression studies, our dataset size is modest, and model generalization must be tested on larger and more diverse cohorts. As noted in recent surveys, EEG-based models sometimes suffer from small sample sizes and heterogeneous protocols. Additionally, we used only EEG data; integrating other modalities (e.g. MRI, clinical surveys, genetic or demographic data) could further enhance accuracy and clinical applicability. Existing research demonstrates that sophisticated multi-path feature fusion (18) techniques enable deep integration of heterogeneous data (19). In future work, we plan to extend HHT-SANN on additional datasets and modalities. Furthermore, the mobile/wearable deployment aligns perfectly with our ongoing efforts to optimize the framework for real-time EEG applications, a priority for future translational research aimed at point-of-care utility.

Conclusions: In conclusion, this study presents HHT-SANN, a novel self-attention neural network that embeds the Hilbert–Huang Transform as a network layer for EEG-based depression screening. By leveraging the adaptive, nonlinear time-frequency decomposition capabilities of HHT alongside attention mechanisms, the proposed model achieves superior classification performance, with 98.78% accuracy, 99.23% sensitivity, and 98.27% specificity. SHAP-based analysis reveals that HHT-derived features contribute over three times more than original EEG signals, highlighting their critical role in decision-making. These results demonstrate that HHT-SANN not only improves diagnostic accuracy but also enhances interpretability, offering a powerful and practical tool for advancing mental health diagnostics through neuroinformatics.

## Data Availability

The original contributions presented in the study are included in the article/supplementary material. Further inquiries can be directed to the corresponding authors.
